# Effect of Collector Rotational Speed on the Morphology and Structure of Solution Blow Spun Polylactic Acid (PLA)

**DOI:** 10.3390/polym16020191

**Published:** 2024-01-09

**Authors:** Nataša Nikolić, Dania Olmos, Ana Kramar, Javier González-Benito

**Affiliations:** 1 Department of Materials Science and Engineering and Chemical Engineering, Universidad Carlos III de Madrid, 28911 Leganés, Madrid, Spain; nnikolic@ing.uc3m.es (N.N.); dolmos@ing.uc3m.es (D.O.); akramar@ing.uc3m.es (A.K.); 2 Instituto Tecnológico de Química y Materiales “Álvaro Alonso Barba”, Universidad Carlos III de Madrid, 28911 Leganés, Madrid, Spain

**Keywords:** solution blow spinning, nanofibers, morphology, image analysis

## Abstract

Apart from structure and composition, morphology plays a significant role in influencing the performance of materials in terms of both bulk and surface behavior. In this work, polylactic acid (PLA) constituted by submicrometric fibers is prepared. Using a modified electrospinning (ES) device to carry out solution blow spinning (SBS), the fibrillar morphology is modified, with the aim to induce variations in the properties of the material. The modification of the ES device consists of the incorporation of a source of pressurized gas (air) and a 3D-printed nozzle of our own design. For this work, the morphology of the PLA submicrometric fibers is modified by varying the rotational speed of the collector in order to understand its influence on different properties and, consequently, on the performance of the material. The rotational speed of a cylindrical collector (250, 500, 1000 and 2000 rpm) is considered as variable for changing the morphology. Morphological study of the materials was performed using scanning electron microscopy and image analysis carried out with ImageJ 1.54f software. Besides a morphology study, structural characterization by Fourier transformed infrared spectroscopy using attenuated total reflectance of prepared materials is carried out. Finally, the morphology and structure of produced PLA fibrous mats were correlated with the analysis of mechanical properties, wettability behavior and adhesion of DH5-α *E. coli* bacteria. It is of interest to highlight how small morphological and chemical structure variations can lead to important changes in materials’ performance. These changes include, for example, those above 30% in some mechanical parameters and clear variations in bacterial adhesion capacity.

## 1. Introduction

Materials used for applications such as membranes, filters, scaffolds, and wound dressings require certain features, for instance, mechanical consistency, biocompatibility, biodegradability, and a particular microstructure or morphology, to satisfy their expected performance. Materials that are constituted by submicrometric materials or nanofibers are able respond to these requirements due to their specific morphology, size and potential orientation [1]. The morphology and topography of materials can highly influence their final properties. These properties include, among others, mechanical properties [2], wettability [3,4], adhesion, and a differentiation of the cells can be affected [5]. A particular case is represented by fibrous materials, as fibers have a high aspect ratio which can lead to a higher impact on the final morphology and even on the topography from, for example, the preferential orientation of the fibers [6].

Well controlled architectures and morphologies can be achieved by electrospinning. It has been stated that the orientation of electrospun material may have a profound effect on cells [7,8]. In addition, one study by Zhao et al., showed that PLA nanofibers with immobilized hyaluronic acid are more efficient against cancer cells than disordered nanofibers [9].

**Figure 1:**
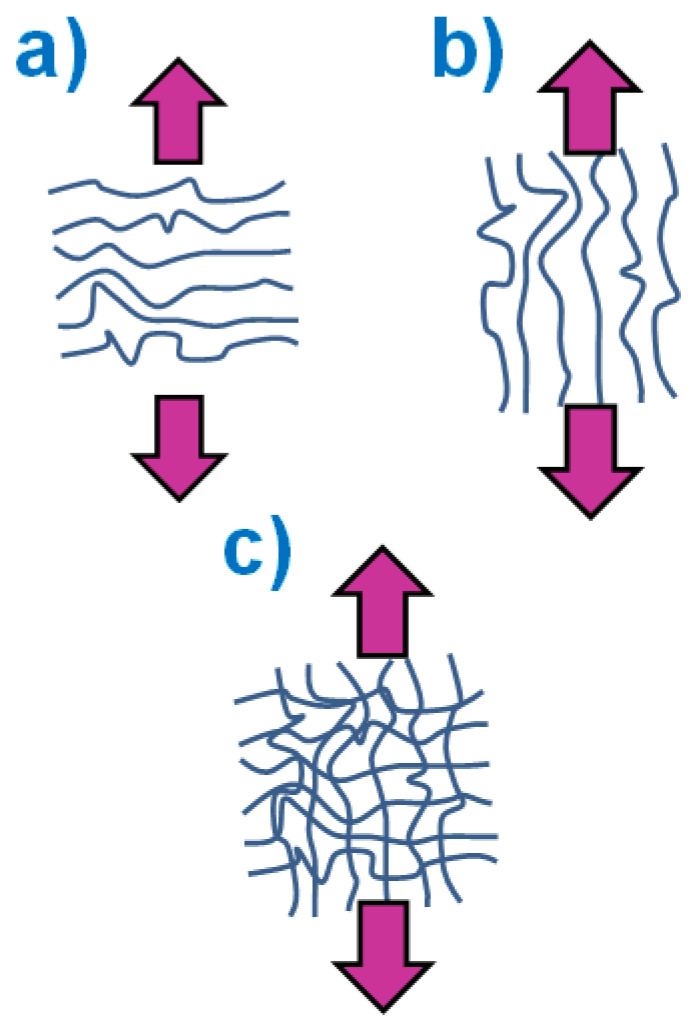
Different configurations of fibers in fibrous materials with respect to the application of load in a tensile test. (**a**) Parallel, (**b**) perpendicular, and (**c**) without preferential orientation.

Fibrillar morphology can also affect mechanical properties. This occurs mainly as a result of two contributions: firstly, the size and shape of the fibers and, secondly, the preferential orientation of the fibers, such as those schematically represented in Figure 1.

We can clearly realize how the direction of application of mechanical loads may affect the material response. When a load is applied parallel to the direction of the long axis of the fibers, better transmission of loads onto fibers is expected and therefore higher mechanical resistance. In fact, M. Lorente et al. have recently showed this behavior in the particular case of polyethylene oxide fibrous materials [2]. Besides mechanical properties and cell growth [10], fiber morphology and orientation can affect the wettability of materials. Several works have showed how morphology can affect the wettability behavior of materials. For instance, in the particular case of polystyrene, it has been reported that, when the material is prepared with a clear flat surface, the water contact angle is around 90°; however, when its morphology arises from submicrometric fibers it is possible for the same polymer to show superhydrophobic behavior even with water contact angles that are clearly higher than 150° [11].

Currently, several methods have been described to prepare materials which allow for the achievement of well-controlled architectures and morphologies that in turn are sometimes even able to mimic the structures of living tissues [8]. Among the methods by which to prepare materials constituted by submicrometric fibers, electrospinning (ES) has, as already mentioned, gained popularity. ES allows for the obtaining of highly porous materials that are mainly constituted by fibers that may have diameters below 100 nm. These nanofibers can mimic extracellular matrices, such as those that can be found in the dermic layer of the skin, and, as a consequence, it is expected they can stimulate cells favoring tissue regeneration [12]. In summary, ES is based on the interaction between a polymer solution emerging from a capillary and a high electric field when applying a potential between the capillary and a collector. As a consequence of the interaction, the polymer solution can be stretched to form fibers while the solvent is evaporating. Although it has been demonstrated that, after choosing adequate ES processing conditions, quite homogeneous materials can be obtained in terms of morphology, certain concerns affect the final use of ES for certain purposes. For example, relatively low production rates are obtained as, in most cases, the solution feeding rate needs to be low. Furthermore, the necessity of a high electric field also introduces difficulties to the process. This is because not all of the systems can interact properly with it to overcome the force exerted on the solution. Because of this, several researchers have more recently been paying attention to another method, known as solution blow spinning (SBS) [13,14,15,16,17,18]. In SBS a polymer solution is injected with the aid of a syringe. A stream of a polymer solution is stretched and projected onto a collector by the action of a pressurized gas. The process is similar to electrospinning, although, in this case, the driving force for stretching the solution is pressurized gas instead of an intense electric field, which makes it more versatile for the preparation of materials. Moreover, solution blow spinning has the potential to be used in situ [19], for example, during a surgical procedure applied directly to the body or a wound, which can be convenient for its use in the preparation of biomaterials.

As has been mentioned, regardless of the nature of a material, microstructure can significantly affect the final properties. In the general case of porous materials, the size, shape and number of pores are important microstructural characteristics to consider. These are factors that, in the particular case of fibrous materials, are directly related with the size, concentration and preferential orientation of fibers. Besides, when extrapolating this microstructure to the surface of the materials important consequences can also be expected. Because of the high aspect ratio, when fibrillar microstructures are considered for scaffolds cells [20] there is also a lot of interest in controlling the orientation of microfeatures. In particular, it is known that the regeneration of highly ordered structures such as those associated with tendons and ligaments can only be carried out effectively in supports (“scaffolds”) that can provide a topographic guide for cells [20]. In fact, since extracellular matrices are formed by organized collagen fibers, many studies have been carried out on the effect of fiber alignment obtained by electrospinning on several types of cells [21,22]. The existence of an intense electric field in ES may be a factor favoring the preferential orientation since the stretching of the solution should occur following the field lines; however, as it was mentioned above the necessity of using an electric field might be a disadvantage. Due to this, other methods such as SBS also able to produce fibers should be investigated.

A few works have focused on the use of SBS [23] and ES [24,25] to obtain fibrous materials where their constituted fibers show preferential orientations. However, the reported distributions of fibers orientations may be confusing since the analysis is usually based on the use of very complicated methods of image analysis [24,25] which results depends on the previous preparation of the image (brightness, contrast, etc.) and on the values of the parameters required by the model to carry out the analysis. Some other works studied the preferential orientation from the simple use of ImageJ software through a manual analysis, by directly measuring the angle associated to the position of the long axis of the fibers on the images obtained by SEM [26,27]. In a work carried out by Jian Zhong et al. in 2015, highly aligned electrospun material was obtained, and reported angles corresponding to the nanofibers’ orientation were low and showing monomodal distribution [26]. In another work by Li, Y et al. 2021, ImageJ analysis of manually measured fibers showed that in ES, the increase of rotational speed of the collector up to 2000 RPM, enables obtaining aligned nanofibers, while with rotational speeds of collector around 300 RPM, orientation was random [27]. Therefore, it is reasonable to think that there is still a necessity of using easy and comprehensible methods of image analysis to study morphology quantitatively or at least semi-quantitatively.

In this work, polylactic acid materials are prepared using a modified electrospinning device to carry out solution blow spinning with the intention of obtaining morphologies constituted by submicrometric fibers. The main aim of the work is to modify the morphology of the PLA in order to understand its influence on different properties and, as a consequence, on the performance of the material. As the unique variable used to modify the morphology, the rotational speed of a cylindrical collector is considered (250, 500, 1000 and 2000 rpm). Morphological and structural characterization of the SBS prepared PLA materials is carried out looking for their relationship with mechanical and wettability behavior, as well as with the adhesion and development of DH5-α *E. coli* bacteria.

## 2. Materials and Methods

### 2.1. Materials

To be processed by solution blow spinning (SBS), polylactic acid (PLA) supplied by Resines Spain SL and manufactured by Nature Works LLC (biopolymer 7032D, δ = 1.24 g·cm^−3^) was used. The polymer data sheet says that the PLA has a glass transition temperature, melting point and crystallization temperature of T_g_ = 55–60 °C, T_m_ = 200–220 °C and T_c_ = 155–175 °C, respectively. As a solvent for the PLA, dichloromethane (DCM) supplied by Merk-Sigma-Aldrich (PCode: 102049248 y CAS: 75-09-2) was used of HPLC or higher quality (boiling point, T_b_ = 39.8–40.0 °C). Magnesium nitrate hexahydrate, Mg(NO_3_)_2_·6H_2_O, (Labkem, Barcelona, Spain) was used to prepare a saturated solution for the control of the relative humidity at 55% (20 °C) in the chamber where the solution blow spinning process is being carried out.

### 2.2. Samples Preparation

**Figure 2:**
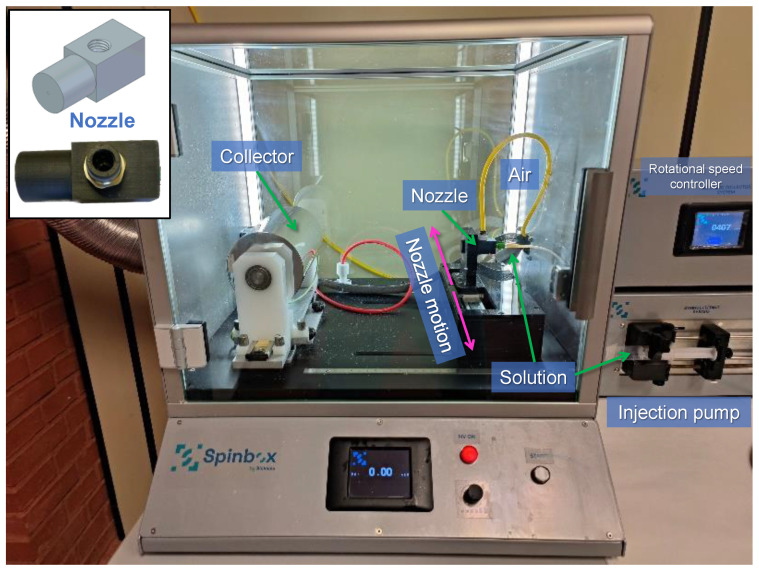
Photograph of the device used to prepare the materials by solution blow spinning.

The PLA solutions were prepared by dissolving 1.1 g of PLA in 10 mL of DCM having solutions of PLA 11% (*w*/*v*). After that, the polymer solutions were processed by solution blow spinning using an electrospinning commercial device (Spinbox Systems^®^, designed by Bioinicia, Valencia, Spain) modified with the incorporation of a 3D printed nozzle designed by our group and a supplying system of pressurized air (Figure 2).

The PLA solution was injected with a syringe at a feeding rate, FR = 0.25 mL/min, in a capillary (inner and outer diameters 0.6 mm and 0.9 mm, respectively) which constitutes the inner channel of the concentric nozzle and that protrudes from the nozzle 2 mm. Pressurized air (2 atm) was simultaneously passed through the outer channel of the nozzle (diameter 1.2 mm). When the solution comes out from the nozzle the pressurized air pulls it and helps the solvent to evaporate, forming the fibers that were collected on a rotating cylindrical collector located at a working distance (WD) of 13 cm. The collector was a solid cylinder covered with aluminum foil where the material was deposited. As the collector rotates at a particular rotational speed (RS) of 250, 500, 1000, or 2000 rpm, the nozzle moves parallel to the long axis of the cylindrical collector from side to side at a constant rate to ensure thickness homogeneity of the collected nonwoven mats. Besides, the materials were prepared at room conditions of 20–25 °C, 1 atm of pressure, and a relative humidity of 55% set by the use of a saturated solution of ammonium nitrate. In the Table 1, the codes used for each sample depending on the rotational speed of the collector and representative photographs of the materials are shown.

### 2.3. Bacteria Culturing

A DH5α strain of the bacteria *E. coli* was cultured on the materials under study following the protocol already used elsewhere [28].

### 2.4. Equipment and Data Analysis

#### 2.4.1. Morphological Characterization

The morphology of the materials was inspected by field emission scanning electron microscopy using a TENEO field emission scanning electron microscope (FESEM-FEI) using an electron acceleration voltage of 10 kV. Samples were glued on SEM specimen’s stubs using double adhesive carbon tape. To make them conductive, specimens were gold coated for 60 s using a Leica EM ACE200 low-vacuum coater. The FESEM images were obtained from the signal arising from backscattered electrons (BSE) detected by a circular backscatter detector (CBS).

To better visualize the *E. coli* bacteria by FESEM they were prepared by treating the samples in the wells used for culturing. 1 mL of glutaraldehyde 2.5 wt% was added to each well to fix and kill bacteria on the materials upon its action for 30 min at room temperature. After that, the glutaraldehyde was removed, and samples were rinsed 3 times with phosphate-buffered saline solution (PBS) to remove the remaining glutaraldehyde. After fixation, the samples were dehydrated in four steps of 10 min where the concentration of ethanol in weight percent was increasing (30, 50, 70 and 100%). Finally, the ethanol was removed leaving the samples in a laminar flow hood to ensure their complete drying.

Images were analyzed using the free software ImageJ. Digital images obtained by FESEM were imported by the ImageJ 1.54ft (Wayne Rasband & contributors, National Institute of Health, Bethesda, MD, USA). With the tool “Measure”, diameters and orientation of fibers were determined. In the first case, from edge to edge of a fiber a segment perpendicular to its major axis is drawn and the corresponding measurement is made using the software tool “Measure”; at least 200 measurements were done on each sample. In the second case, a segment of at least 10 μm is drawn along the major axis of a fiber. Apart from giving the length of the segment drawn, the ImageJ tool “Measure” gives the orientation of the segment in degrees (°) in the column header “Angle”; in this case, at least 200 measurements were also done. Size and orientation of *E. coli* bacteria were also measured following the same protocol used to analyze fibers. For bacteria, segments corresponding to the whole length of bacteria were used to determine their orientations.

#### 2.4.2. Structural Characterization

Structure of the materials was studied by infrared spectroscopy using a Nicolet iS5 spectrometer (Thermo scientific, Thermofisher, Madison, WI, USA) equipped with an attenuated total reflectance (ATR) device with diamond window GladiATR (PIKE Technologies, Madison, WI, USA). The spectra were collected in the middle range from 400 to 4000 cm^−1^ from the average of 32 scans and with a resolution of 4 cm^−1^.

#### 2.4.3. Porosity Studies

The porosity of the samples was determined by gravimetry. At least three square-shaped specimens were cut from the samples and carefully dimensioned by measuring thickness with the use of a micrometer (Digimatic, Mitutoyo Corporation, Barcelona, Spain, with an accuracy of ±1 μm) and the length and width by the use of a caliper (±0.01 mm accuracy). The final size of each specimen was taken as the mean thickness, length and width from at least 5 measurements of each dimension. From the product of mean thickness, length and width the volume of each specimen is calculated. Then, the specimens are weighted out to finally obtain the densities of the specimens considered from the quotient between the mass and the volume. Final density of the sample, *δ_s_*, is calculated as an average from the densities of the specimens. If porosity is defined as the percentage of volume occupied by air in the material, *%V_air_* (Equation (1))

(1) $$Porosity=\%V_{air}=\frac{V_{air}}{V_{T}}\cdot 100\%$$

where *V_air_* is the volume of the sample occupied by air and *V_T_* is the total volume of the sample or the volume occupied by the polymer plus de volume occupied by the air. Therefore, it is easy to demonstrate that the porosity is one minus the quotient between the density of the sample (apparent density) and the density of the material in bulk (*δ* = 1.24 g·cm^−3^ information given by the supplier), without pores and multiplied by one hundred (Equation (2)).

(2) $$Porosity=\%V_{air}=\left(1-\frac{\delta_{s}}{\delta }\right)\cdot 100\%$$

#### 2.4.4. Wettability Studies

Wettability was studied by measuring water contact angles using the sessile drop method on an OCA-15 Plus Goniometer (Data Physics, Neurtek Instruments, Eibar, Spain). Drops of distilled and deionized water of about 3 μL were dispensed on the surface of the films. After less than 5 s photographs of the drops were taken to make the water contact angle measurement on them. The results were expressed as an average from 5 measurements per sample.

#### 2.4.5. Mechanical Testing

The Mechanical behavior was studied using a Universal testing machine Microtest DT/005/FR (Microtest S.A., Madrid, Spain) with a load cell of 50 N. Specimens were tested in a uniaxial tensile configuration using a loading rate of 5 mm/min. The dimensions of the specimens were 40 mm in length, 10 mm in width, and the gap between the grips was 20 mm. Mechanical tests were done 5 times for each sample using two sets of specimens. The first set corresponded to those cut parallel to the direction of the collector rotation during SBS (parallel specimen, ‖) and the second set corresponded to those specimens cut perpendicular to the direction of the collector rotation during SBS (perpendicular, ⊥). Specimens’ thickness was measured before each test using Digimatic micrometer (Mitutoyo Corporation, Barcelona, Spain) of ±1 μm accuracy.

## 3. Results and Discussion

**Figure 3:**
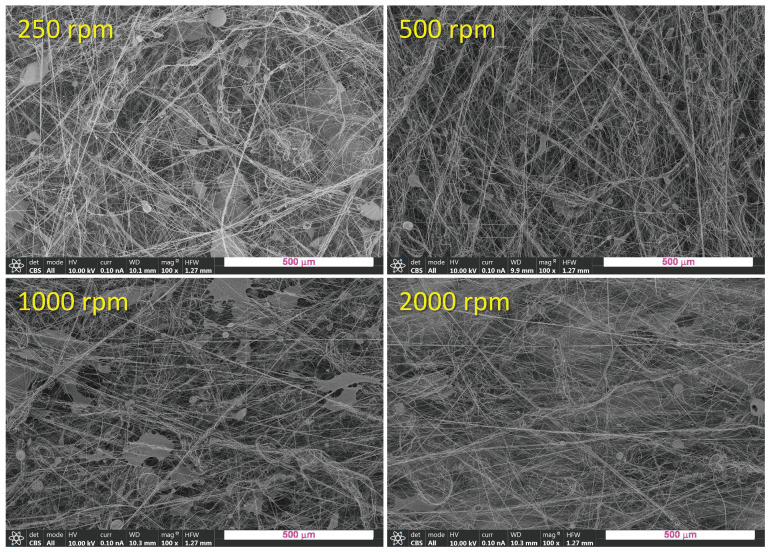
FESEM images of the PLA mats obtained at different rotational speeds of the collector at a magnification of 100× (scale bar 500 μm).

In the Figure 3 FESEM images of the PLA mats obtained at different rotational speeds of the collector are shown at a magnification of 100×. All the materials are mainly constituted by fibers showing quite similar morphology where differences in microfeatures cannot be observed, probably because of the relatively low magnification used. In order to have quantitative information arising from the morphology, higher magnification was used and a careful image analysis was carried out by the use of the free software ImageJ.

**Figure 4:**
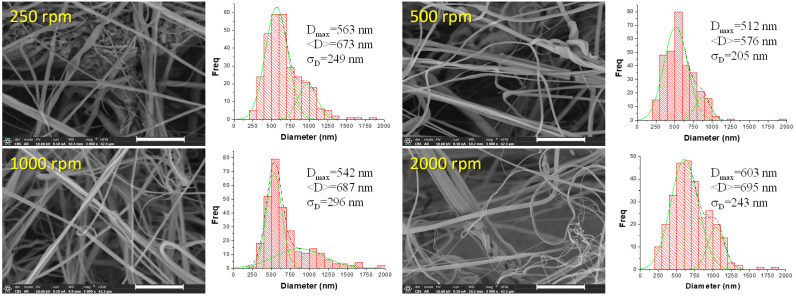
FESEM images of the PLA mats obtained at different rotational speeds of the collector are shown at a magnification of 3000× and distributions (bar histograms) of fibers sizes in terms of their diameters fitted by multi-Gaussian functions (green lines, convoluted into the dashed black lines). White scale bar refers to 10 μm.

In the Figure 4, FESEM images obtained at higher magnification for the PLA mats are shown together with distributions (bar histograms) of fiber sizes in terms of their diameters. In order to better visualize the diameter distributions, they were fitted by multi-Gaussian functions (green dashed lines). As can be seen, all the materials are constituted by submicrometric fibers with diameters ranging from 100 nm to 2000 nm. Although the size of the fibers is very similar regardless of the rotational speed of the collector used, there is a slight decrease in the diameters of fibers and in the width of the diameters distribution when the collector RS increases from 250 rpm to 1000 rpm. Then, at 2000 rpm, the average diameter and the width of the distribution increase. These observations can be confirmed by extracting several parameters from the diameter distributions such as the average of the distribution or the average diameter, *<D>*, the diameter with maximum probability, *D_max_*, and the standard deviation, *σ_D_* (Table 2).

Although differences are small, a possible explanation of the results obtained can be the consideration of an extra fiber drawing because of longitudinal stress exerted by the rotating collector when the fiber is attached to it. In principle, it is expected to exert more stress on the fiber using higher rotational speed and that is what is observed, until 1000 rpm. However, in the case of 2000 rpm, the distribution of diameters is more heterogeneous (wider distribution) and, on average, the size of the fibers increases. One possible reason to explain this result can be the strong interaction existing between the air and the surface of the collector which can create uncontrolled currents of air. The formation of a kind of turbulences might avoid proper attachment of the fibers on the collector without being able to exert the corresponding stress considered above.

**Figure 5:**
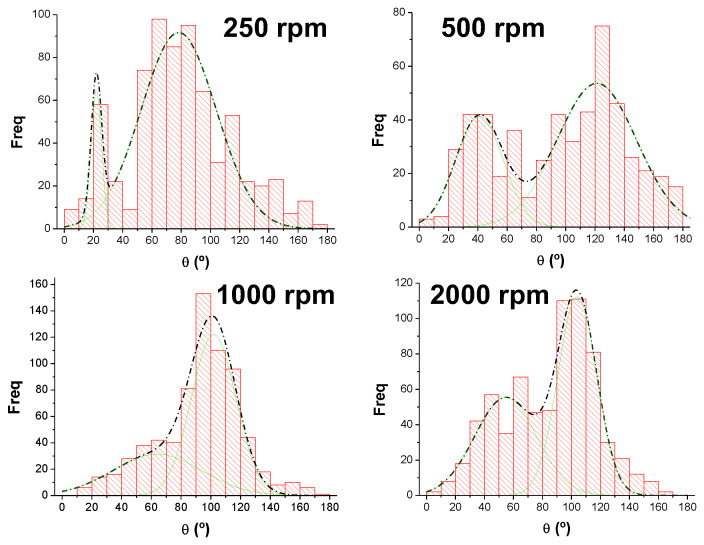
Distributions of angles (bar histograms) corresponding to the orientations of fibers for the four materials collected at different rotational speeds and their corresponding multi-Gaussian fitting (green lines, convoluted into the dashed black lines).

On the other hand, the orientation of the fibers was also analyzed with ImageJ. In general, distributions of angles corresponding to different orientations as those shown in Figure 5 are obtained. As can be seen, these distributions were bimodal. These bimodal distributions can be explained by considering the way of obtaining the materials. When the SBS device is working, the nozzle is continuously moving following a perpendicular direction to that of the collector rotation. For this reason, when the nozzle is moving to one side, it is expected to have a preferred orientation of the fibers while, when it is moving to the other side it is expected to have another preferred orientation for them [29]. Therefore, one would expect that the difference between two preferred orientations were lower as the higher rotational speed is used because each time the fibers collection should be more controlled by the collector rotation. Distributions shown in the Figure 5 almost confirmed what was expected.

In general, it is observed that the higher the rotational speed the more oriented the fibers (each time the maxima of the two preferential orientations associated with the bimodal distribution are closer or less distinguished from each other). However, at 2000 rpm a wider distribution than the one corresponding to 1000 rpm is obtained. This result can be explained considering that too high rotational speed of the solid collector may cause air currents that may avoid proper collection of fibers, leading to more random deposition of fibers on the collector.

**Figure 6:**
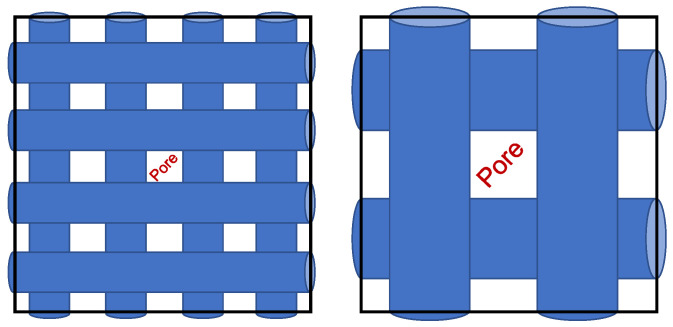
Scheme representing the relationship between size of fibers, porosity and the size of pores.

Another parameter highly related to the morphology is the porosity estimated in this work by the use of the Equation (2). In the last column of the Table 2 the values of porosity obtained for the different materials under study are gathered. Although all the values are very close to each other it can be observed that there is a trend for which the porosity increases as the rotational speed of the collector increases. This result is again in accordance with the idea that the faster the rotation speed the more difficult the fiber attachment to the collector. Finally, if porosity is considered together with the general trend observed for the size of the fibers, it can be concluded that, at least from 500 rpm, the size of pores increases as the higher the rotational speed is. The Figure 6 shows models which, visually demonstrate for the same amount of material constituted by fibers, how the thicker the fibers are the larger the pores if they are considered to be randomly distributed. These models consider 2D materials constituted by 2D fibers represented by rectangles.

**Figure 7:**
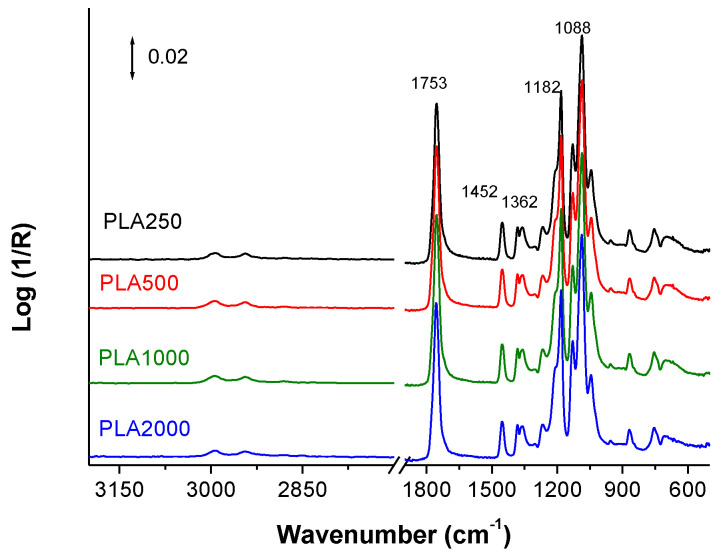
ATR-FTIR spectra of the PLA mats obtained at different rotational speeds of the collector.

Regarding the structure, FTIR spectra of the prepared materials are shown in Figure 7. Typical bands of PLA appear always in the same position: the absorption of carbonyl groups, C=O, at 1753 cm^−1^, the absorption corresponding to bending of methyl groups –CH_3_ (antisymmetric at 1452 cm^−1^ and symmetric at 1361 cm^−1^) the absorption corresponding to the stretching vibrations of C–O groups at 1188 cm^−1^ (symmetric) and at 1088 cm^−1^ (antisymmetric) [30]. As can be seen, there is not any variation in the position of the absorption bands or in the relative contribution of them respect to the whole spectra of PLA. Therefore, the chemical characteristics of the surfaces should be the same and consequently performance in terms of, for example, wettability or adhesion of bacteria is not expected to change due differences of specific interactions but as a consequence of other causes such as for example, changes in the morphology.

**Figure 8:**
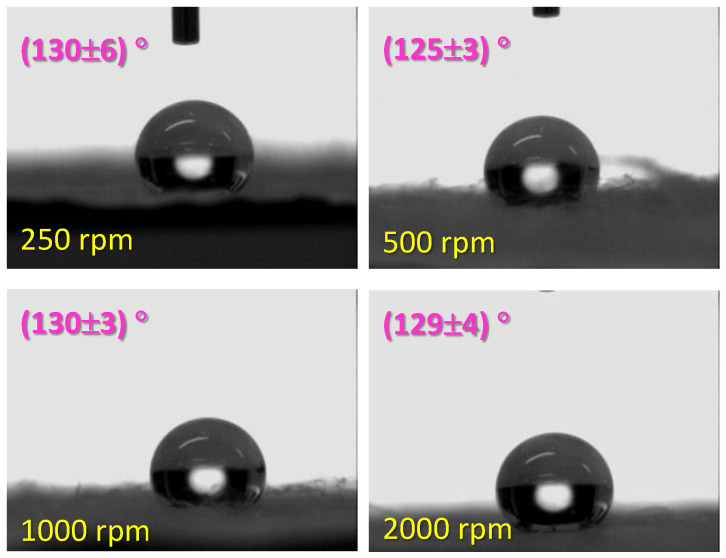
Photographs of water drops on the PLA mats prepared by SBS and their corresponding contact angles.

In order to study the wettability, water contact angles were measured. As can be seen in the Figure 8, all materials presented the similar water contact angle within the range of 125–130°. These results point out that when PLA is prepared in the form of nonwoven mats it behaves quite more hydrophobic than when PLA is prepared in the form of flat solid films with water contact angles lower than 80° [31,32,33]. This result can be explained considering that the higher the PLA surface available, the lower the water contact angle. On the other hand, if the error is not considered, it might be said that the sample collected at 500 rpm yielded the lowest contact angle that only can be correlated with the size of fibers and correspondingly with the size of pores. It seems therefore that, in the particular case of PLA, thinner fibers lead to lower water contact which can also be explained by considering that more of surface is available for the water to wet the material [34]. Considering the two well-known models used to describe the wettability behavior of solid surfaces, Wenzel [35], and Cassie-Baxter [36], and the explanation given by J.E. Domínguez et al. [34] it can be concluded that wettability behavior of the PLA system under consideration is better explained by the Cassie-Baxter approximation [37], which states that the relation between the real contact angle formed on the rough surface, *θ_CB_*, and the contact angle formed on the smooth surface, *θ*, is given by

*cos θ_CB_* = *φ_S_* × *cos θ* + *φ_S_* − 1 (3)

where *φ_S_* is the fraction of solid surface in direct contact with the liquid (surface available to water) which increases with the lowering of the contact angle, in accordance with our results.

**Figure 9:**
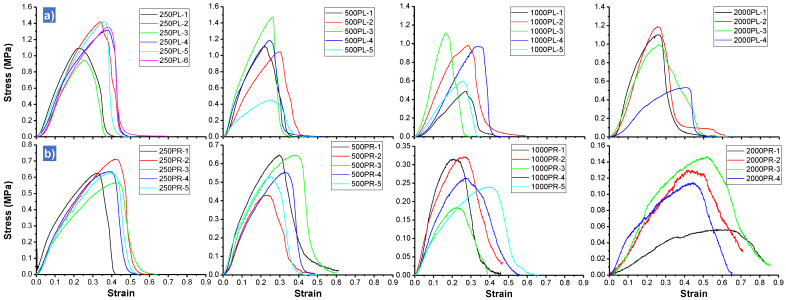
Strength-strain curves for all the specimens tested: (**a**) parallel specimens, ‖ and (**b**) perpendicular specimens, ⊥. Strain is given in (1/1).

The mechanical behavior of the prepared materials was studied by tensile tests. Figure 9a (parallel specimens, ‖) and Figure 9b (perpendicular specimens, ⊥) show the stress-strain curves for all the specimens tested. In general, all the plots’ profiles are similar, where a sharp increase of strength can be found at low strains, then, maximum strength is smoothly reached and finally, during the failure of the material, a decrease in stress is observed without being abrupt but rather gradual. This later result seems to be similar to those found for fibrous materials and fiber reinforced composites [38,39]. This behavior can be explained considering that failure does not only occur as a consequence of bulk material failure but also by mechanical disentanglement process. This step of fibers disentanglement can occur sequentially if there is not a homogeneous transfer of mechanical loads as would be expected for this type of systems in which to a greater or lesser extent the fibers are entangled with each other.

From here, two variables are considered for the analysis of results; the first one is the rotational speed of the collector and, the second one, the direction for the specimen preparation for mechanical tests, parallel or perpendicular, respectively. Let us start with specimens whose long axis is coincident with the direction of the rotation of the collector (parallel, ‖). To carry out the analysis different mechanical parameters were chosen, maximum strength, *σ_max_*, Young’s modulus, *E*, resilience, *U_y_*, toughness in terms of total energy absorbed along the tensile test, *U*, and width at half height, *W*_._

**Figure 10:**
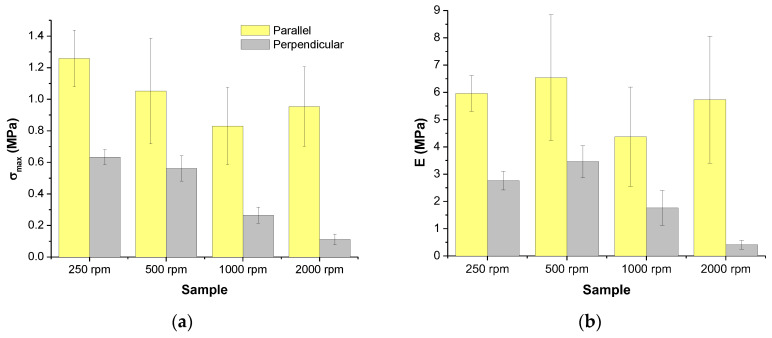
Tensile strength or maximum strength (**a**) and Young’s modulus (**b**) of PLA-based materials obtained by SBS at different rotational speeds of the collector.

In the Figure 10, the tensile strength or maximum strength is represented as a function of rotational speed for both kinds of specimens, parallel and perpendicular. As can be seen, lower tensile strengths than 1.5 MPa were obtained which is quite lower than the one obtained for PLA, 50–60 MPa [40,41]. This result can be easily explained considering that the section used to calculate the stress is one arising from the dimensions of the specimens, therefore, the section was over-estimated since the materials prepared in this work are highly porous.

When comparing the type of specimen during mechanical tests, it is observed that for the specimens that have their long axis parallel to the direction of the collector rotation, the mechanical strength is higher. This result can be explained considering that the long axis of the fibers is preferentially oriented to the long axis of the specimens which should imply better transmission of mechanical loads since more points of interactions between the fibers are possible. On the other hand, for both kinds of specimens it is observed a general trend, the higher the rotational speed the lower the strength. Considering that structural changes were not observed (Figure 7), only morphological variations must be the cause of this general behavior. In the present work, three morphological parameters were considered, size of the fibers, preferential orientation of fibers and porosity. As can be seen, when relating results shown in Figure 10 with data gathered in Table 2, only the porosity could be correlated with mechanical strength. When porosity increases, the mechanical strength decreases as expected. Considering that when the porosity increases, the real section that supports the loads is smaller; in other words, the section used to calculate the stress was overestimated.

From the elastic behavior of the specimens, the Young’s modulus is extracted and the mean values are represented in the Figure 10. Again, it seems that due to the porosity lower modulus for PLA was obtained [40,41]. On the other hand, as in the case of the tensile strength, rigidity represented by the Young’s modulus is higher for the specimens with their long axis parallel to the direction of the rotation of the collector which can also be explained by the consideration of a better transmission of loads along longer fibers. However, when considering the rotational speed of the collector as the variable we cannot see any clear trend. These results can be explained considering there is not any relationship between morphology (size of the fibers, porosity, and preferential orientation of fibers) variations observed and the elastic modulus.

However, there exists the possibility that changes in modulus are not clearly observed because of the small variations in the parameters describing the morphology. In the case of the specimens cut in perpendicular direction to the rotation of the collector a slight trend can be observed (Figure 10). It seems that the higher the preferential orientation, the lower the Young’s modulus.

**Figure 11:**
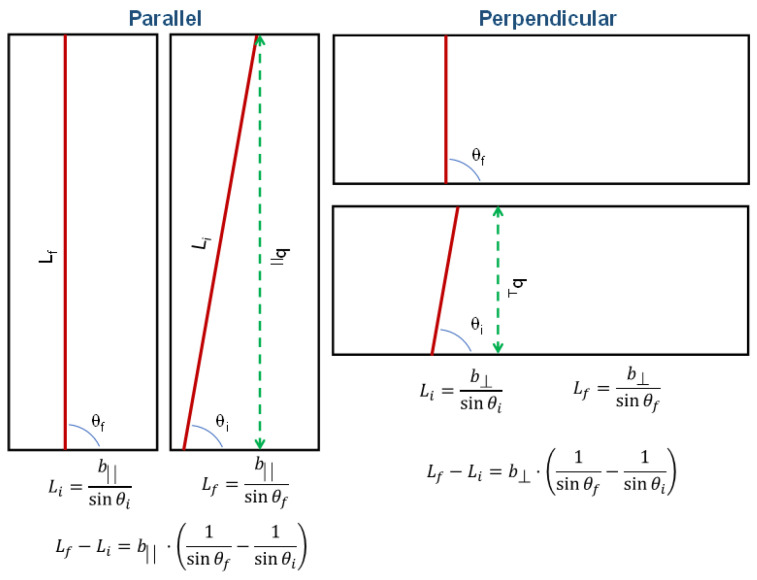
Models of specimens (parallel and perpendicular) form the representation of only one fiber as a straight line with a certain length, the specimens would be constituted by many other equal fibers.

For the specimens perpendicular it is expected to have shorter fibers (see the scheme represented in Figure 11 where models with only one representative fiber are shown as a straight line with a certain length. Even though the specimens are made up of many equally-sized fibers, small changes in the orientation of these fibers will have a bigger impact on load transmission. This is because variations in the average lengths of the fibers will be greater for these small changes in orientation. This reason can be demonstrated by trigonometry defining the length decrease of fibers, *L_f_* − *L_i_*. Here, *L_i_* represents the initial length of the fiber in the specimen and *L_f_* represents the final length after changing the preferential angle of orientation. Considering that all specimens have the same dimensions (Figure 11) the initial length of the fibers can be expressed by:

(4) $$L_{i}=\frac{b}{\sin\theta_{i}}$$

where *b* is the side opposite and *θ_i_* is the initial angle associated to the fibers’ orientation (Figure 11), therefore, the change in length would be:

(5) $$L_{f}-L_{i}=b\cdot \left(\frac{1}{\sin\theta_{f}}-\frac{1}{\sin\theta_{i}}\right)$$

Taking into account that the side opposite in the parallel specimen (*b*_‖_) is longer than the side opposite in the perpendicular specimen (*b*_⊥_), it can be said that the reduction in fiber length is less pronounced as they become more oriented in relation with the direction of collector rotation in the case of perpendicularly cut specimens. Consequently, a higher impact of orientation on modulus changes is expected for the perpendicular specimens. In perpendicular specimens, preferred orientation of fibers doesn’t favor the transmission of load. When fibers are more oriented in this direction lower modulus are expected, considering that the decrease in length is minimal. However, in the case of parallel specimens, the decrease of length might be significant enough to balance the positive contribution of the orientation respect to the transmission of loads.

**Figure 12:**
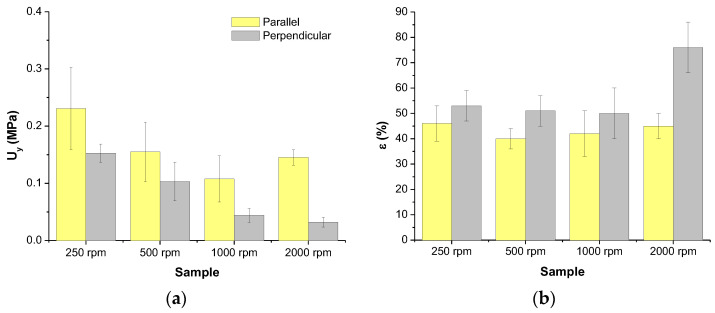
Modulus of resiliency (**a**) and Elongation at break at σ = 0 (**b**) of PLA-based materials obtained by SBS at different rotational speed of the collector.

Another interesting mechanical property is the resiliency represented by the parameter modulus of resiliency, *U_y_*, the energy absorbed by the material up to yield. Figure 12 shows the values of *U_y_* of the materials under study. It is observed that the materials are elastically tougher when the tensile load is applied parallel to the direction of rotation of the collector. Besides, regardless of the orientation of the specimens, the higher the rotation speed of the collector the lower the resiliency. These results can be explained through the same reason used to explain the tensile strength; in fact, resiliency is mainly correlated with that parameter rather than the Young’s modulus.

Regarding the elongation at break, *ε*, it can be observed in the Figure 12 that, in general, it is larger when the load is applied perpendicularly to the direction of the collector rotation. This result can be explained considering that strain of the specimens should imply generation of more entanglements when fibers tend to be aligned with the direction of application of load. We could explain the mechanism of strain as constituted by two steps: (1) orientation of fibers and (2) slippering of fibers. Therefore, when perpendicular specimens are considered both steps should have an important contribution to the strain. However, when the load is applied in the same direction as that of the rotation of the collector, if the fibers are also mainly oriented to that direction, only the contribution of the second step (slippering of fibers) should be the predominant one, and less strain is expected. On the other hand, it can also be observed that the faster the rotation of the collector the higher the elongation at break, which might be related to the preferred orientation of fibers.

**Figure 13:**
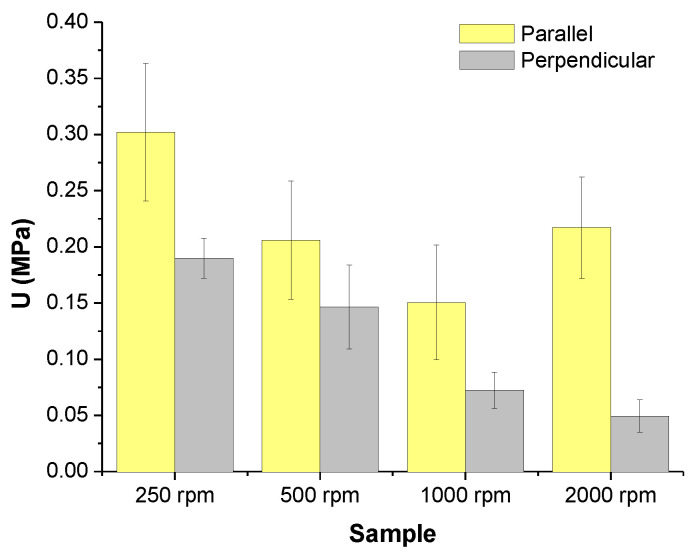
Energy absorbed at break of PLA-based materials obtained by SBS at different rotational speeds of the collector.

When considering the total energy, *U*, absorbed by the materials along the whole tensile test (Figure 13, toughness) the same trends as those obtained for the resiliency were obtained (Figure 12). Although elongation at break should favor the toughness, for the materials under consideration the tensile strength is the important factor accounting for the toughness. Therefore, the same reasons given to explain the tensile strength can be used to justify the results obtained for the energy absorbed at break during the tensile test.

**Figure 14:**
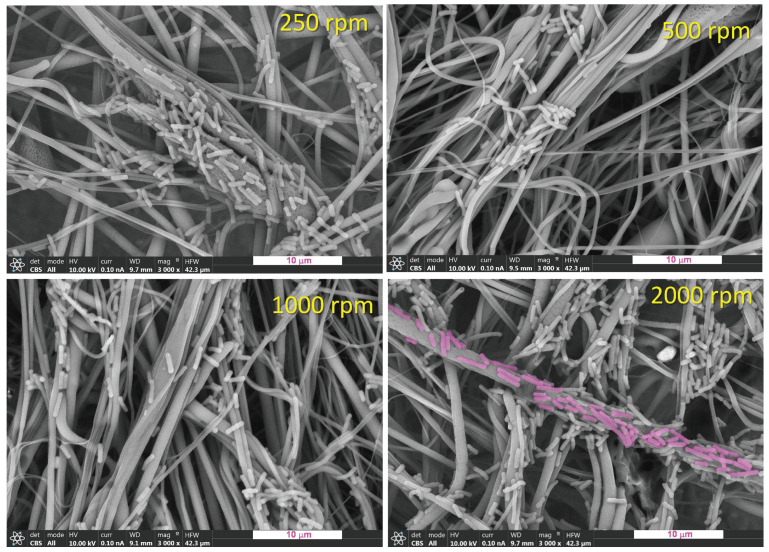
Representative images of bacteria attached to the materials prepared at different rotation speeds of the collector (scale bar 10 μm).

**Figure 15:**
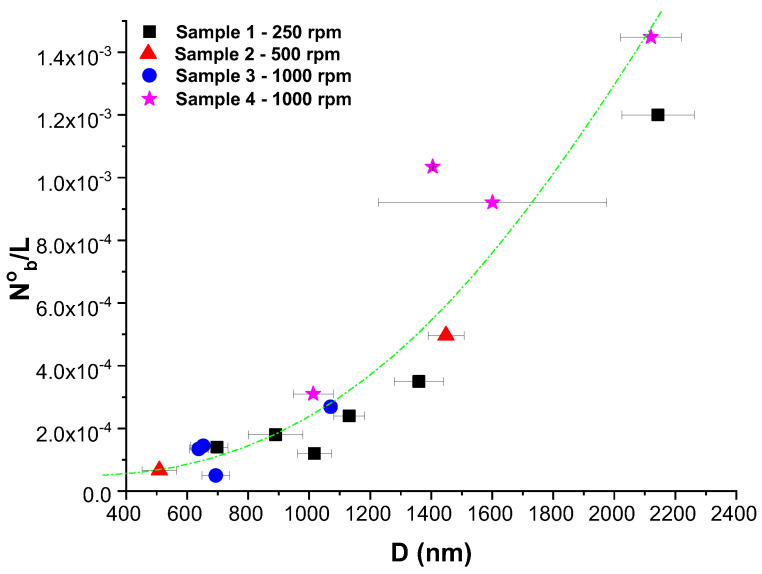
Number of bacteria per unit of surface are as a function of the average diameter associated to the fibers.

Finally, a study of the influence of the morphology on the adhesion and development of bacteria was carried out. A strain DH5-α of *E. coli* was cultured on the materials and after fixing them an inspection by SEM was carried out. As can be seen in the Figure 14, bacteria are directly attached to the fibers and, when a fiber is thick enough bacteria seems to follow the same orientation as the fiber. Besides, it seems that the thicker the fiber the more bacteria are attached. To see this better, bacteria were counted per unit of surface area and represented as a function of the average diameter of the fibers in particular sample (Figure 15). As can be seen, there is a clear trend that confirms that the higher the diameter the higher the number of bacteria attached to the material. Therefore, one important conclusion is that in order to avoid bacteria proliferation, one factor is to control the size of the fibers trying them to be as thinner as possible.

On the other hand, as can be seen in Figure 14, the size of pores or the empty space in between fibers is large enough as the tiny bacteria *E. coli* can escape through them. Therefore, if the fibers do not offer surface enough as the bacteria to be properly attached there won’t be bacteria adhesion. Another different thing would be the possibility of other kind of cells to be adhered, which would depend on their sizes and their possibility to be attached to the material if they can lie on several fibers.

**Figure 16:**
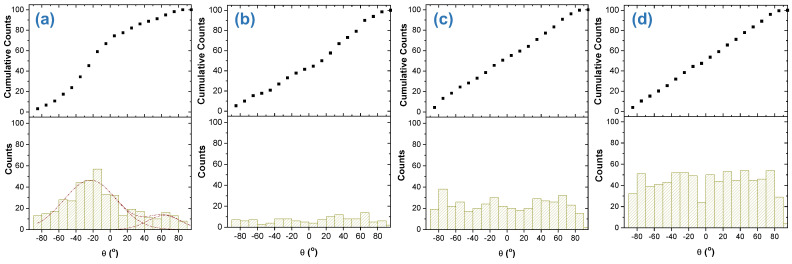
Distribution of orientations for the bacteria attached to the materials under study: (**a**) 250 rpm; (**b**) 500 rpm; (**c**) 1000 rpm and (**d**) 2000 rpm. (Cumulative counts were normalized to 100).

Another important issue to consider is the possibility of orienting cells preferentially by the preferred orientation of the microfeatures associated to a particular morphology. In this work, DH5-α *E. coli* bacteria were chosen as model cells for this purpose. In the Figure 16 it can be clearly seen a preferential orientation of bacteria when they are attached to fibers that are thick enough. Therefore, in order to achieve a preferential orientation of cells, there must be first a preferred orientation of fibers and second, they must be thick enough. To see if there is any correlation between the preferential orientation of fibers in a sample with the orientation of bacteria in the Figure 16 it is shown the distribution of orientations for the bacteria. As can be seen, there is only preferential orientation of bacteria for the material collected at 250 rpm. The reason for that is because analysis of bacteria adhered only could be done on a few fibers thick enough. For the rest of the materials, it could not be observed any preferred orientation of bacteria when only one fiber is considered and when it is thick enough.

## 4. Conclusions

In this work, the influence of rotational speed of the collector during solution blow spinning on the size and orientation of the fibers was studied, as well as a potential correlation between morphology induced by different speeds of collector rotation and properties of PLA nanofibrous material. This investigation revealed that the increase of rotational speed of the collector increases the porosity of materials (from 79% to 83%), slightly increases the average diameter of fibers as well as the diameter of fiber with maximum probability in distribution. The lowest fiber size was obtained when 500 rpm was used. The orientation of fibers was represented by bimodal distribution (due to the horizontal movement of the nozzle during processing) and with increasing rotational speed, the maxima of both distributions were closer to each other, suggesting more oriented fibers. However, increase of rpm to 2000, caused less orientation in fibrous materials, probably because too high speed of the collector may cause turbulent air currents that prevent proper orientation of fibers, leading to a more random deposition. Tensile strength decreases when rotational speed of the collector increases, as well as modulus of resiliency and total energy absorbed by fibers during testing, which can be considered as a consequence of porosity. Opposite to this, strain at break is increasing with an increase of rotational speed of the collector, and moreover it is higher for specimens that were cut perpendicular to the rotational direction. The study of bacterial adhesion revealed that number of bacteria that adhere per unit of surface is increasing with increasing size of fibers, i.e., bacteria will easily adhere to thicker fibers. Therefore, to prevent bacterial adhesion onto the surface of fibrous material, it is favorable to have thinner fibers. This conclusion can have a significant impact on the decision for final application of a certain material, whereby when designing materials for potential biomedical application, the attention should be given to the morphology and size of nanofibers as well.

## Figures and Tables

**Table 1:** Sample codes associated to the rotational speeds of the collector and representative images of the materials prepared.

Sample Code	Rotational Speed (rpm)	Image
PLA250	250	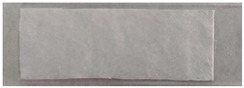
PLA500	500	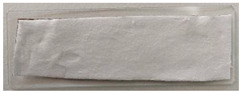
PLA1000	1000	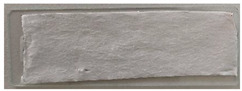
PLA2000	2000	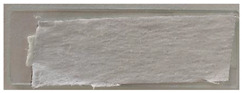

**Table 2:** Morphological parameters extracted from the FESEM image analysis.

Sample	*D_max_* (nm)	*<D>* (nm)	*σ_D_* (nm)	*Δθ* (°)	*<θ>* (°)	*σ_θ_* (°)	* *θ_max_* (°), (Width, Area)	Porosity (%)
PLA250	563	673	249	56	80	35	22, (7, 577) 78, (51, 5908)	79.5 ± 0.4
PLA500	512	576	205	80	97	44	41, (32, 1667) 121, (55, 3684)	81.8 ± 1.2
PLA1000	542	687	296	49	93	28	51, (30, 1295) 100, (32, 5506)	82.2 ± 1.5
PLA2000	603	695	243	49	85	31	55, (43, 2976) 104, (27, 3768)	83.6 ± 1.0

* Two *θ_max_* (°) correspond to the two Gaussian peaks.

## Data Availability

Data are contained within the article.
